# Knockdown of circ_0002194 protects against oxidized low-density lipoprotein-induced cell damage via the regulation of the miR-637/PACS2 axis in human vascular endothelial cells

**DOI:** 10.1093/icvts/ivac210

**Published:** 2022-08-11

**Authors:** Ruyang Mei, Mei Wu, Fei Ren

**Affiliations:** Department of Cardiovascular Medicine, First People's Hospital of Jingmen, Jingmen, Hubei, China; Department of Gastroenterology, First People's Hospital of Jingmen, Jingmen, Hubei, China; Department of Cardiovascular Medicine, First People's Hospital of Jingmen, Jingmen, Hubei, China

**Keywords:** CircRNA, MIRN637, PACS2, Oxidized LDL, Atherosclerosis

## Abstract

**OBJECTIVES:**

Atherosclerosis is one of the most common cardiovascular diseases. The functional roles of circular (circ) RNAs have been discovered in atherosclerosis. Our goal was to explore the regulation and mechanism of circ_0002194 in oxidized low-density lipoprotein-induced human vascular endothelial cells.

**METHODS:**

Circ_0002194, microRNA-637 (miR-637) and phosphofurin acidic cluster sorting protein 2 (PACS2) levels were determined through the reverse transcription-quantitative polymerase chain reaction. Cell viability was detected using the Cell Counting Kit-8 assay, and angiogenetic ability was analysed via the tube formation assay. Flow cytometry was used to measure cell apoptosis. Western blot was performed to examine protein expression. Oxidative stress was assessed using commercial kits. The RNA immunoprecipitation assay and dual-luciferase reporter assay were conducted for target analysis.

**RESULTS:**

Treatment with oxidized low-density lipoprotein induced the upregulation of circ_0002194 in endothelial cells. Cell viability and angiogenesis were promoted while cell apoptosis and oxidative stress were reduced by the downregulation of circ_0002194 in the cell model. Furthermore, miR-637 was identified as an miRNA target of circ_0002194, and the regulatory role of circ_0002194 was associated with the sponge effect on miR-637. Moreover, circ_0002194 could regulate PACS2 by affecting miR-637. Additionally, miR-637 suppressed endothelial cell damage by partly mediating the expression of PACS2.

**CONCLUSIONS:**

The results demonstrated that circ_0002194 facilitated endothelial cell dysfunction in atherosclerosis partly through upregulating PACS2 by targeting miR-637.

**Figure:**
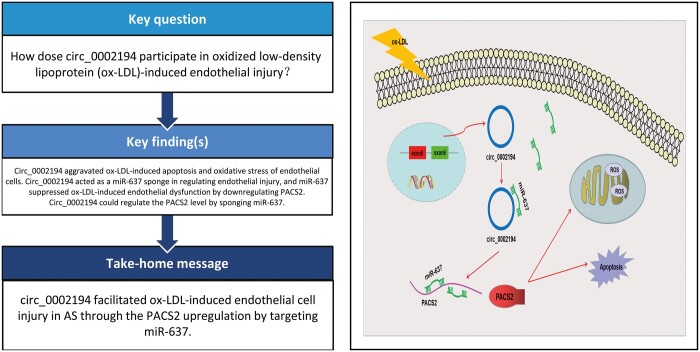

## INTRODUCTION

Atherosclerosis (AS) is an arterial disease with chronic systemic inflammation [1]. The functional and structural integrity of the endothelium is important in maintaining vascular homeostasis and preventing AS [2]. Endothelial dysfunction acts as a high-risk factor for the incidence of AS [3]. In addition, oxidized low-density lipoprotein (ox-LDL) is an oxidizing agent leading to the abnormal function of endothelial cells in the atherosclerotic process [4]. Importantly, exploring the molecular mechanism of endothelial cell dysfunction will provide more perspectives for predicting and treating AS.

Noncoding circular RNAs (circRNAs) have special covalently closed-loop structures with high stability. Most circRNAs can function as microRNA (miRNA) sponges to alter the gene expression levels [5]. CircRNAs are associated with the pathogenic processes underlying the progression of AS, including endothelial injury [6]. Li *et al.* found that silencing circ_0068087 relieved the ox-LDL-induced oxidative and inflammatory damage to endothelial cells by reducing the ROBO1 level through mediating miR-186-5p [7]. Additionally, circ_0000345 protected against cell apoptosis in ox-LDL-stimulated endothelial cells by affecting the miR-129-5p/TET2 axis [8], and circ_0124644 enhanced endothelial dysfunction in AS by mediating the miR-149-5p/PAPP-A axis [9].

Circ_0002194 is an exonic circRNA from exon 5 and exon 6 of RELT-like 1 (RELL1) with a mature length of 376 base pairs. A recent study has indicated that circ_0002194 facilitated the endothelial inflammation induced by ox-LDL through sponging miR-6873 to upregulate MyD88 expression [10]. However, the other regulatory mechanism of circ_0002194 in AS is still unknown. The short miRNAs are also implicated in the development of AS, and microRNA-637 (miR-637) has diagnostic or prognostic values in patients with AS [11, 12]. Moreover, phosphofurin acidic cluster sorting protein 2 (PACS2) has been shown to promote ox-LDL-induced endothelial cell apoptosis [13]. The online prediction indicated the potential target relation between circ_0002194 and miR-637 as well as miR-637 and PACS2. Thus, the association among circ_0002194, miR-637 and PACS2 in AS deserves further investigation.

**Figure 1:**
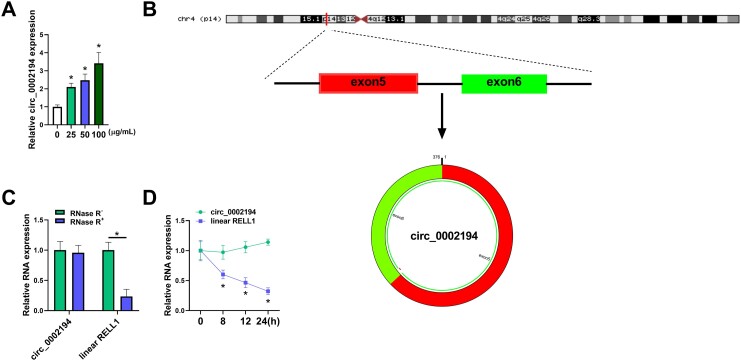
Treatment of oxidized low-density lipoprotein upregulated the level of circular (circ)_0002194 in human umbilical vein endothelial cells (HUVECs). (**A**) The circ_0002194 level was examined using reverse transcription-quantitative polymerase chain reaction after the HUVECs were treated with 0 μg/ml, 25 μg/ml, 50 μg/ml or 100 μg/ml oxidized low-density lipoprotein. (**B**) The genic information of circ_0002194. (**C-D**) The levels of circ_0002194 and RELT-like 1 were quantified by the reverse transcription-quantitative polymerase chain reaction after RNA was treated with RNase R (**C**) or HUVECs were incubated with actinomycin D (**D**). *P <* *0.05. circ: circular; RELL1: RELT-like 1.

This study hypothesized that circ_0002194 could modulate the PACS2 level by targeting miR-637. Furthermore, the functional assays were conducted to determine whether the regulatory role of circ_0002194 was related to the miR-637/PACS2 axis in the endothelial injury of AS.

## MATERIALS AND METHODS

The current study was approved by the ethical review committee of First People's Hospital of Jingmen. Written informed consent forms were signed by all patients.

### Cell culture and oxidized low-density lipoprotein treatment

Human umbilical vein endothelial cells (HUVECs) (BeNa, Beijing, China) were cultured in F-12K medium (BeNa) with the complement of 10% fetal bovine serum (BeNa) and a 1% penicillin/streptomycin solution (100 ×, BeNa). After incubation in a 37°C and 5% CO_2_ incubator overnight, cells were treated with increasing concentrations (0 μg/ml, 25 μg/ml, 50 μg/ml and 100 μg/ml) of ox-LDL (Sigma, St. Louis, MO, USA) for 24 h [9].

### Transient transfection

After treatment with 50 μg/ml ox-LDL for 24 h, HUVECs were transfected with oligonucleotides via a Lipofectamine 3000 Kit (Invitrogen, Carlsbad, CA, USA). Negative control (NC) or circ_0002194 of small interfering (si) RNA (si-NC, si-circ_0002194#1/#2/#3), mimic negative control or miR-637 mimic (mimic NC, miR-637 mimic), negative control or miR-637 of an inhibitor (inhibitor NC, miR-637 inhibitor), and negative control or PACS2 of siRNA (si-ctrl, si-PACS2) were provided by RIBOBIO (Guangzhou, China). These RNAs were diluted in Opti-MEM reduced serum medium, and added with the Lipofectamine 3000 reagent for 15 min. A total of 60% confluent cells were incubated with the prepared mixture in the culture incubator, followed by cell collection for further use.

### Reverse transcription-quantitative polymerase chain reaction assay

Total RNA extraction (using the TRIzol reagent) was used to isolate RNA from HUVECs; then RNA expression was detected with the One-Step Reverse Transcription-Quantitative Polymerase Chain Reaction (RT-PCR) Kit for reverse transcription and 2 × TaqMan Fast qPCR Master Mix for real-time polymerase chain reaction. These kits were purchased from Sangon (Shanghai, China). Then, an analysis of the relative level of RNA was performed via the 2^-ΔΔCt^ method [14]. The expression was normalized to glyceraldehyde-phosphate dehydrogenase or U6. The stability of circ_0002194 and RELL1 was determined by the reverse transcription-quantitative polymerase chain reaction (RT-qPCR) after RNA was treated with RNase R (Epicentre Technologies, Madison, WI, USA) for 1 h, and HUVECs were incubated with actinomycin D (Sigma) for various times (0 h, 8 h, 12 h, 24 h). All primers (Table 1) were synthesized by Sangon.

**Table 1.** Primer sequences used for the reverse transcription-quantitative polymerase chain reaction

Name	Primer sequences (5’-3’)
circ_0002194	Forward: GAACGGAGAAGCCTGATGTC
	Reverse: ACAAAGGCCCAGGACTCACT
microRNA-637	Forward: GCCGAGACTGGGGGCTTTCG
	Reverse: CTCGTATCCAGTGCAGGGT
RELT-like 1	Forward: TCATGGGTCTCTTTGGCGTC
	Reverse: AGACATCAGCATTCGCTTCA
GAPDH	Forward: AGCCACATCGCTCAGACAC
	Reverse: GCCCAATACGACCAAATCC
U6	Forward: GCTTCGGCAGCACATATACTAAAAT
	Reverse: CGCTTCACGAATTTGCGTGTCAT

GAPDH: glyceraldehyde-phosphate dehydrogenase

### Cell Counting Kit-8 assay

HUVECs were exposed to 50 μg/ml ox-LDL and transfected with different RNAs. Then cells were added with the Cell Counting Kit-8 (Sigma) by 10 μL per well at the indicated time points (0 h, 24 h, 48 h, 72 h). The absorbance examination at λ = 450 nm was carried out 2 h later through the microplate reader (Thermo Fisher Scientific, Waltham, MA, USA).

### Tube formation assay

Angiogenetic ability was assessed according to the number of branches in the HUVECs. The 96-well plates were enveloped with 60 μL Matrigel (BD Bioscience, San Diego, CA, USA) and seeded with 2 × 10^4^ HUVECs. After being cultured for 48 h, capillary-like branches were observed, and the average number was calculated using a computer-assisted microscope.

### Flow cytometry

Cell apoptosis was measured by double staining using the Annexin V Apoptosis Detection Kit (Sangon). A total of 5 × 10^4^ HUVECs were resuspended with 1 × Binding Buffer, followed by incubation with 5 μL Annexin V-fluorescein isothiocyanate and 10 μL propidium iodide (PI) away from light. After staining for 20 min, a cell analysis was conducted under the flow cytometer (BD Biosciences). Annexin V+/PI- and Annexin V+/PI+ labelled cells were considered to be the apoptotic cells.

### Western blot assay

The protein level analysis was performed by Western blot as previously described [15]. After protein extraction by radioimmunoprecipitation assay lysis buffer (Sigma), 50 μg of protein of each sample was used for subsequent detection. The primary antibodies included anti-Bcl-2 (Abcam, Cambridge, UK; ab32124, 1:1000), anti-Bcl-2 associated X (anti-Bax; Abcam, ab32503, 1:1000) and anti-PACS2 (Thermo Fisher Scientific, PA5-72866, 1:1000). Goat anti-rabbit immunoglobin G (IgG) H&L antibody (Abcam, ab205718, 1:5000) was used as the secondary antibody to combine with the primary antibody. Then, protein blots were exhibited by a highly sensitive enhanced chemiluminescence reagent (Sangon), and protein levels were analysed via the ImageJ software (NIH, Bethesda, MD, USA).

### Oxidative assay

A total of 3 × 10^4^ treated HUVECs were collected to determine the level of oxidative injury. The reactive oxygen species (ROS) and malondialdehyde (MDA) levels were detected according to the instruction books of the Total Reactive Oxygen Species Assay Kit (Invitrogen, Thermo Fisher Scientific, Waltham, MA) and the Lipid Peroxidation (MDA) Assay Kit (Sigma).

### RNA immunoprecipitation assay

The Imprint RNA Immunoprecipitation Kit (Sigma) was used to analyse the interaction between circ_0002194 and miR-637. HUVECs were incubated with the antibody-coated magnetic beads at 4°C overnight. Antibody against IgG served as the negative control for the Argonaute-2 protein. RNA mixtures on the beads were acquired, followed by circ_0002194 and miR-637 detection using RT-qPCR.

### Dual-luciferase reporter assay

The bioinformatics analysis between targets was conducted through CircInteractome (https://circinteractome.nia.nih.gov/) and Targetscan (http://www.targetscan.org). The circ_0002194 wild-type (wt) sequence (with miR-637 binding sites) and the mutant-type (mut) sequence (with mutated miR-637 sites) were amplified for constructing the luciferase plasmids (circ_0002194 wt, circ_0002194 mut) using the pmirGLO plasmid (Promega, Madison, WI, USA). In addition, PACS2 three prime untranslated region (3'UTR) wt and PACS2 3'UTR mut plasmids were obtained for the binding analysis between PACS2 and miR-637. The 293 T cells (BioVector NTCC Inc., Beijing, China) were cultured in Dulbecco’s modified eagle medium containing 10% FBS, then co-transfected with wt or mut plasmids and mimic NC or miR-637 mimic for 48 h. A Dual-luciferase Reporter Detection Kit (Promega) was applied to determine the luciferase activity of each group.

### Statistical analyses

The current data were collected from 3 replicates and manifested as the mean ± standard deviation. Then, the data were processed through SPSS 22.0 (SPSS Inc., Chicago, IL, USA) and the statistical difference (*P *<* *0.05, significant) was analysed using the Student *t*-test or analysis of variance followed by Tukey’s test.

## RESULTS

### Treatment of oxidized low-density lipoprotein upregulated the level of circ_0002194 in human umbilical vein endothelial cells

HUVECs were exposed to different concentrations of ox-LDL for 24 h. The detection of the expression by RT-qPCR showed that circ_0002194 was upregulated in 25 μg/ml, 50 μg/ml or 100 μg/ml groups relative to the 0 μg/ml group (Fig. 1A). Circ_0002194 is produced by back-splicing of exon 5 and exon 6 of RELL1 gene, and its mature length is 376 bp (Fig. 1B). To compare the stability of circ_0002194 and linear RELL1, RNase R and actinomycin D treatments were performed in RNA and in cells respectively. The results demonstrated that circ_0002194 was more stable but that RELL1 was downregulated by RNase R (Fig. 1C) and actinomycin D (Fig. 1D). Thus, ox-LDL induced the upregulation of circ_0002194 in HUVECs.

Silencing circ_0002194 enhanced cell viability and angiogenesis but inhibited apoptotic and oxidative damages in ox-LDL-treated HUVECs.

**Figure 2:**
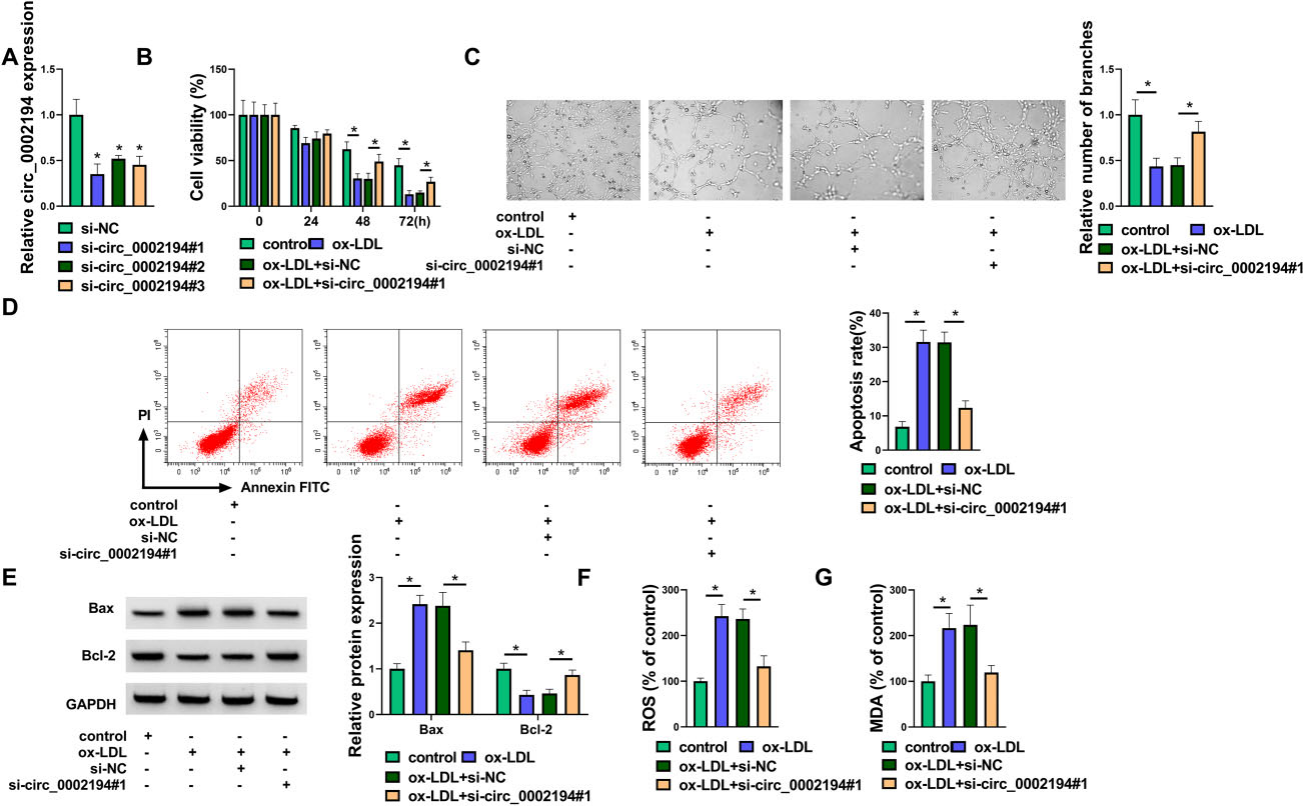
Silencing circular (circ_0002194 enhanced cell viability and angiogenesis but inhibited apoptotic and oxidative damage in oxidized low-density lipoprotein (ox-LDL)-treated human umbilical vein endothelial cells. **(A)** The reverse transcription-quantitative polymerase chain reaction was used to assess the knockdown efficiency of small interfering (si) RNAs for circ_0002194. **(B-G)** Human umbilical vein endothelial cells were treated with control, ox-LDL (50 μg/ml), ox-LDL+si-negative controls and ox-LDL+si-circ_0002194#1. **(B-C)** The Cell Counting Kit-8 assay and the tube formation assay were used to determine cell viability **(B)** and angiogenesis **(C)**. **(D)** Flow cytometry was used to measure the cell apoptosis rate. **(E)** The Western blot was used to detect the protein levels of Bax and Bcl-2. **(F-G) (F)** Detection of reactive oxygen species and malondialdehyde levels **(G)** was used to analyse the oxidative injury (*P *<* *0.05). circ: circular; FITC: fluorescein isothiocyanate; GAPDH: oxidized low-density lipoprotein; MDA: malondialdehyde; NC: negative control; OX-LDL: oxidized low-density lipoprotein; ROS: reactive oxygen species; si: small interfering.

The specific siRNAs were used to detect the level of inhibition of circ_0002194. As shown in Fig. 2A, circ_0002194 expression was significantly reduced after transfection of siRNAs (si-circ_0002194#1, si-circ_0002194#2 and si-circ_0002194#3). The subsequent assays were performed using the si-circ_0002194#1 with the highest knockdown efficiency. Cell viability (Fig. 2B) and angiogenetic ability (Fig. 2C) were suppressed by ox-LDL, whereas these effects were eliminated with si-circ_0002194#1 transfection. Flow cytometry analysis showed that cell apoptosis was repressed by silencing circ_0002194 in ox-LDL-treated HUVECs (Fig. 2D). Also, si-circ_0002194#1 reversed the ox-LDL-induced upregulation of Bax (pro-apoptosis marker) and the downregulation of Bcl-2 (anti-apoptosis marker) (Fig. 2E). ROS and MDA levels were decreased in the ox-LDL+si-circ_0002194#1 group compared with the ox-LDL+si-NC group, indicating that circ_0002194 inhibition attenuated the oxidative stress caused by ox-LDL (Fig. 2F-G). These data revealed that ox-LDL-induced cell dysfunction was attenuated after the knockdown of circ_0002194.

### Circ_0002194 could bind to microRNA-637

**Figure 3:**
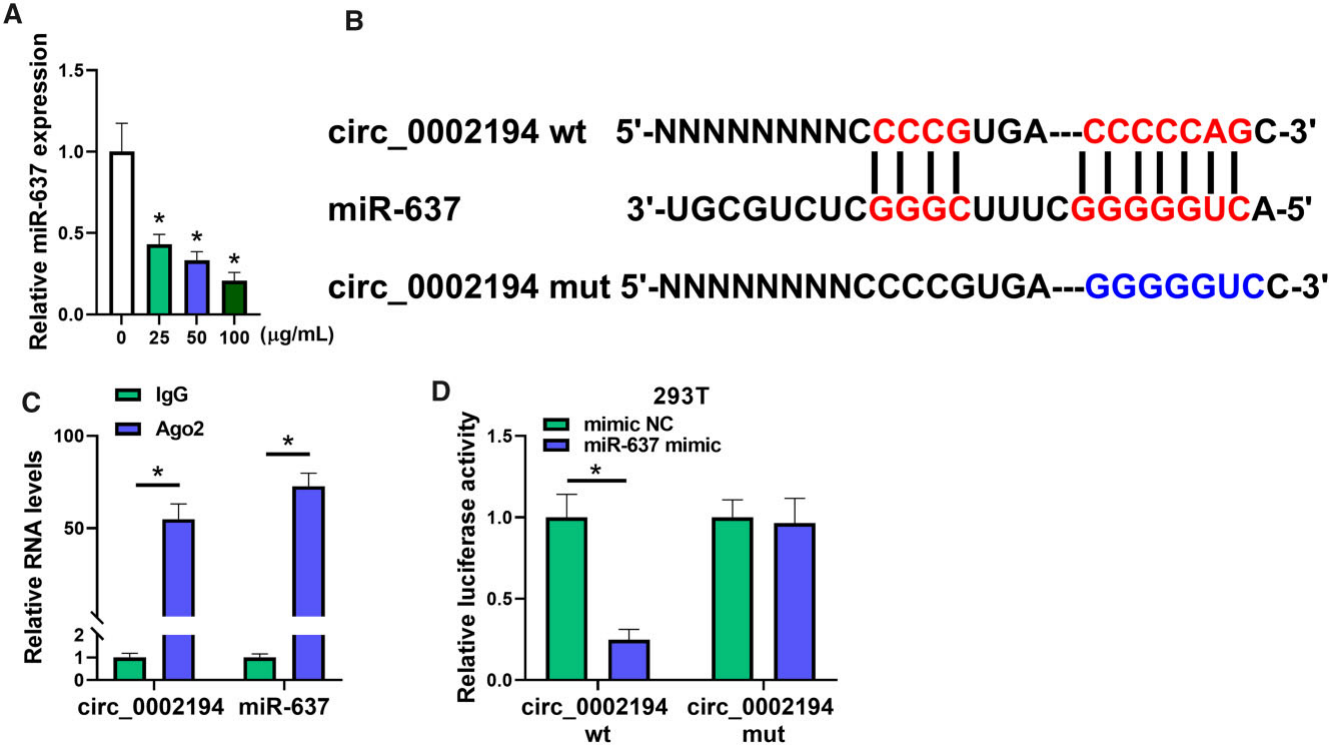
Circ_0002194 could bind to microRNA-637 (miR-637). **(A)** The miR-637 was quantified using reverse transcription-quantitative polymerase chain reaction in 0 μg/ml, 25 μg/ml, 50 μg/ml or 100 μg/ml oxidized low-density lipoprotein treatment groups. **(B)** The binding sites between circ_0002194 and miR-637 were predicted by CircInteractome. **(C-D)** The interaction between circ_0002194 and miR-637 was determined by the Imprint RNA Immunoprecipitation assay in human umbilical vein endothelial cells **(C)** and the dual-luciferase reporter assay in 293 T cells **(D)**. *P <* *0.05. Ago 2: Argonaute-2; circ: circular; IgG: immunoglobin G; miR-637: microRNA-637; mut: mutant-type; NC: negative control; wt: wild-type.

After exposure to ox-LDL, RT-qPCR showed that the level of miR-637 was obviously downregulated in HUVECs compared to the control group (Fig. 3A). The online bioinformatics analysis of CircInteractome showed that circ_0002194 had the binding sites for miR-637 (Fig. 3B). Then, the Imprint RNA Immunoprecipitation assay and the dual-luciferase reporter assay were performed to affirm the target interaction between circ_0002194 and miR-637. The levels of circ_0002194 and miR-637 were increased by the capture of the Argonaute-2 protein, in comparison to the IgG group (Fig. 3C). The co-transfection of miR-637 mimic and circ_0002194 wt resulted in the inhibition of luciferase activity relative to that of the mimic NC+circ_0002194 wt group, but no difference in luciferase activity was detected in the circ_0002194 mut plasmid with mimic NC or miR-637 mimic transfection (Fig. 3D). Overall, circ_0002194 interacted with miR-637 with the miRNA sponging function.

The protective role of circ_0002194 downregulation against ox-LDL was inhibited by the miR-637 inhibitor in HUVECs.

**Figure 4:**
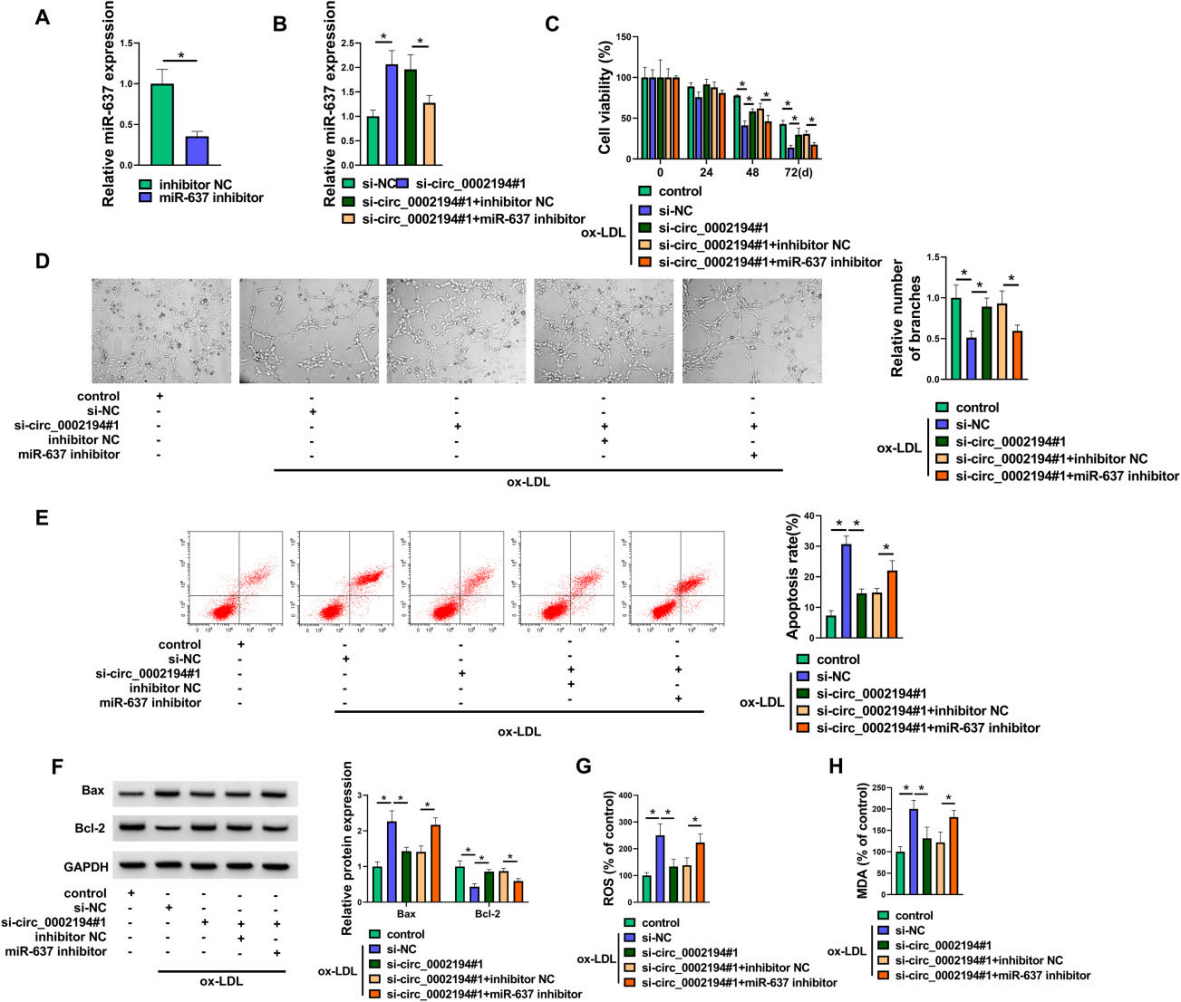
The protective role of circ_0002194 downregulation against oxidized low-density lipoprotein (ox-LDL) was inhibited by the microRNA-637 (miR-637) inhibitor in human umbilical vein endothelial cells (HUVACs). **(A)** The expression of miR-637 was detected by reverse transcription-quantitative polymerase chain reaction in HUVECs transfected with inhibitor NC or miR-637 inhibitor. **(B)** The level of miR-637 was determined using reverse transcription-quantitative polymerase chain reaction after transfection of siNC, si-circ_0002194#1, si-circ_0002194#1+inhibitor NC or si-circ_0002194#1+miR-637 inhibitor. **(C-H)** HUVACs were treated with control, ox-LDL (50 μg/ml) +si-NC, ox-LDL+si-circ_0002194#1, ox-LDL+si-circ_0002194#1+inhibitor NC, ox-LDL+si-circ_0002194#1+miR-637 inhibitor. **(C-D)** The examination of cell viability **(C)** and angiogenesis **(D)** was conducted via the Cell Counting Kit-8 assay and the tube formation assay. **(E-F)** The assessment of cell apoptosis was conducted by flow cytometry **(E)** and Western blot **(F)**. **(G-H)** The evaluation of oxidative stress was conducted via detecting the levels of ROS **(G)** and MDA (H). *P <* *0.05. circ: circular; GAPDH: oxidized low-density lipoprotein; miR-637: microRNA-637; NC: negative control; ox-LDL: oxidized low-density lipoprotein; si: small interfering.

The miR-637 level was repressed in the miR-637 inhibitor group relative to the inhibitor NC group in HUVECs, showing that the miR-637 inhibitor was effective in reducing the expression of miR-637 (Fig. 4A). In addition, the upregulation of miR-637 by si-circ_0002194#1 was counteracted after the transfection of the miR-637 inhibitor (Fig. 4B). The ability of si-circ_0002194#1 to promote cell viability (Fig. 4C) and tube formation (Fig. 4D) but to inhibit the regulation of cell apoptosis (Fig. 4E-F) and oxidative stress (Fig. 4G-H) could be mitigated by the suppression of miR-637 expression in ox-LDL-exposed HUVECs. Altogether, circ_0002194 regulated ox-LDL-induced cell injury by inhibiting miR-637.

### Phosphofurin acidic cluster sorting protein 2 expression was mediated by the circ_0002194/microRNA-637 axis

**Figure 5:**
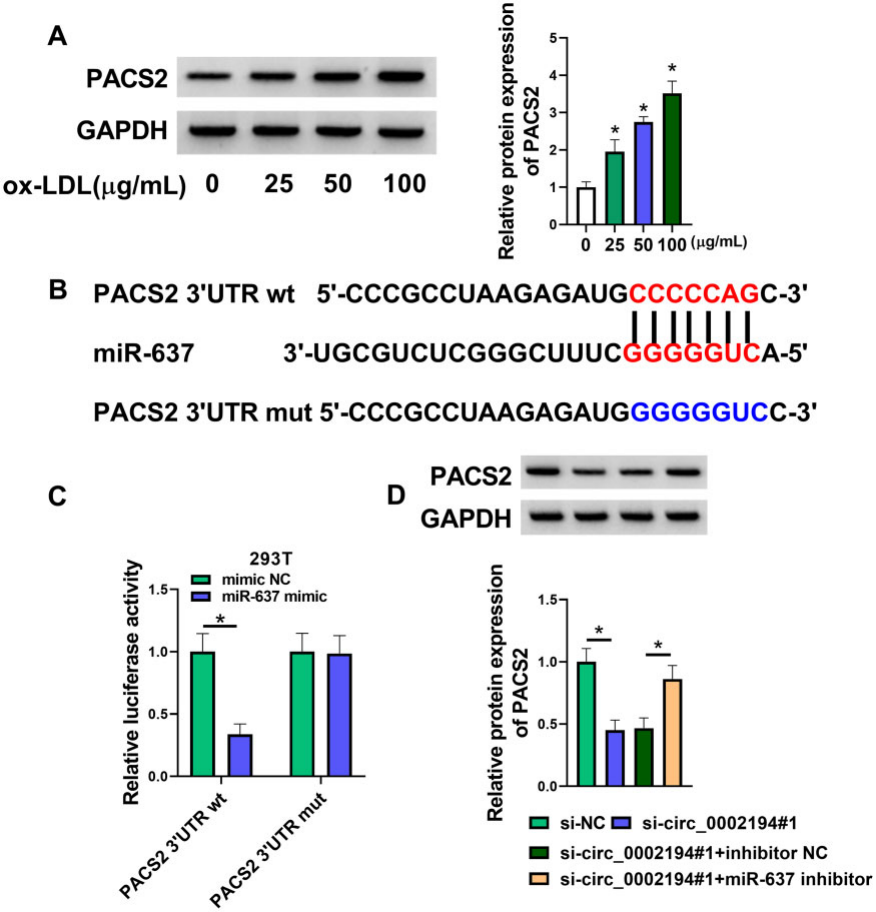
The expression of phosphofurin acidic cluster sorting protein 2 (PACS2) was mediated by the circ_0002194/microRNA-637 (miR-637) axis. **(A)** The PACS2 protein level was measured by the Western blot after treatment with 0 μg/ml, 25 μg/ml, 50 μg/ml and 100 μg/ml oxidized low-density lipoprotein. **(B-C)** The binding between PACS2 and miR-637 was predicted by Targetscan **(B)** and affirmed by the dual-luciferase reporter assay in 293 T cells **(C)**. (D) The Western blot was applied to examine the protein expression of PACS2 in the small-interfering (si)-negative control, si-circ_0002194#1, si-circ_0002194#1+inhibitor negative control or the si-circ_0002194#1+miR-637 inhibitor group. *P <* *0.05. GAPDH: oxidized low-density lipoprotein; miR-637: microRNA-637; mut: mutant-type; NC: negative control; OX-LDL: oxidized low-density lipoprotein; PACS2: phosphofurin acidic cluster sorting protein 2; si: small interfering; 3'UTR: three prime untranslated region; wt: wild-type.

By performing a Western blot assay, we found that PACS2 was highly expressed in ox-LDL-treated HUVECs (Fig. 5A). Interestingly, Targetscan predicted the binding site between the PACS2 3'UTR sequence and the miR-637 sequence (Fig. 5B). Overexpression of miR-637 reduced the luciferase activity of the PACS2 3'UTR wt plasmid rather than the PACS2 3'UTR mut plasmid in 293 T cells, suggesting the target binding between miR-637 and PACS2 (Fig. 5C). Additionally, the miR-637 inhibitor abrogated the si-circ_0002194#1-mediated protein inhibition of PACS2 in HUVECs (Fig. 5D). Circ_0002194 could affect the level of PACS2 by targeting miR-637.

Inhibition of miR-637 aggravated the ox-LDL-induced dysfunction in HUVECs by upregulating PACS2.

**Figure 6.**
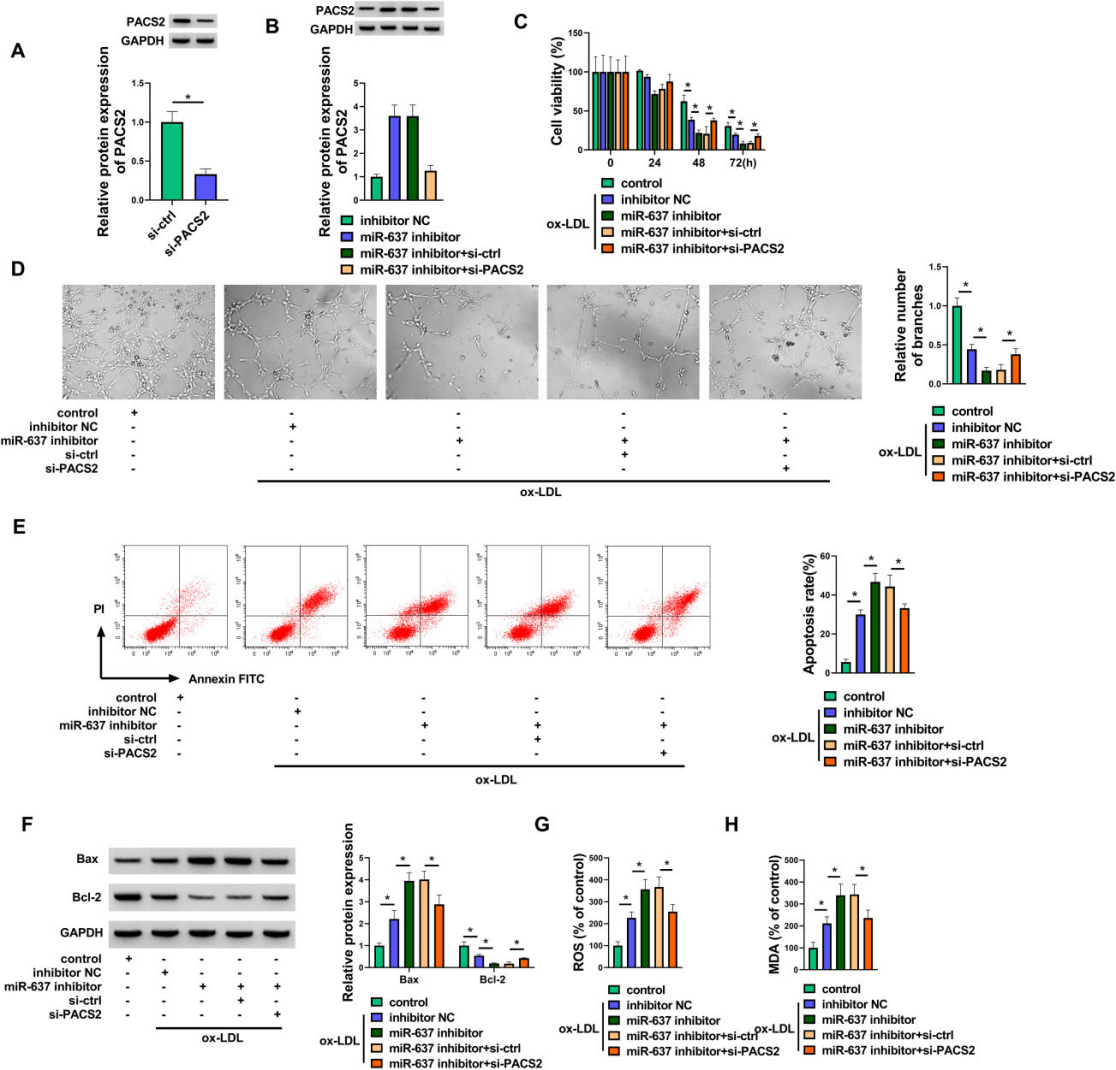
Inhibition of microRNA-637 (miR-637) aggravated the ox-LDL-induced dysfunction in human umbilical vein endothelial cells via upregulating PACS2. **(A)** The protein analysis of PACS2 was carried out by Western blot in si-ctrl or si-PACS2 transfected human umbilical vein endothelial cells. **(B)** PACS2 protein expression was examined by Western blot following transfection of the inhibitor NC, miR-637 inhibitor, miR-637 inhibitor+si-ctrl or miR-637 inhibitor+si-PACS2. **(C-H)** Groups of control, ox-LDL (50 μg/ml) +inhibitor NC, ox-LDL+miR-637 inhibitor, ox-LDL+s miR-637 inhibitor+si-ctrl, ox-LDL+ miR-637 inhibitor+si-PACS2. **(C-D)** Cell viability **(C)** and angiogenesis **(D)** were determined through the Cell-Counting Kit-8 assay and the tube formation assay. **(E-F)** Cell apoptosis was assessed through flow cytometry **(E)** and Western blot **(F)**. (**G-H)** Oxidative stress was measured by examining ROS **(G)** and MDA levels **(H)**. *P <* *0.05. ctrl: control; FITC: fluorescein isothiocyanate; GAPDH: oxidized low-density lipoprotein; MDA: malondialdehyde; miR-637: microRNA-637; NC: negative control; OX-LDL: oxidized low-density lipoprotein; PACS2: phosphofurin acidic cluster sorting protein 2; ROS: reactive oxygen species; si: small interfering.

The effects of miR-637 and PACS2 were further researched in ox-LDL-treated HUVECs. Western blot analysis demonstrated that si-PACS2-induced downregulation of PACS2 levels was conspicuous in HUVECs (Fig. 6A). PACS2 protein expression was upregulated by the miR-637 inhibitor, which was counterbalanced by si-PACS2 (Fig. 6B). The Cell Counting Kit-8 assay and the tube formation assay showed that miR-637 inhibition enhanced the ox-LDL-induced inhibition of cell viability (Fig. 6C) and angiogenesis (Fig. 6D), whereas the knockdown of PACS2 alleviated these regulatory influences. The results of flow cytometry (Fig. 6E) and apoptotic protein detection (Fig. 6F) suggested that cell apoptosis was promoted by the miR-637 inhibitor through the upregulation of PACS2. Examination of ROS and MDA indicated that silencing PACS2 abated the oxidative stress caused by downregulation of miR-637 in ox-LDL-treated HUVECs (Fig. 6G-H). The preceding evidence affirmed that miR-637 could relieve cell damage in ox-LDL-induced HUVECs by the negative regulation of PACS2.

### Circ_0002194/microRNA-637/phosphofurin acidic cluster sorting protein 2 regulated oxidized low-density lipoprotein-induced endothelial cell dysfunction

**Figure 7.**
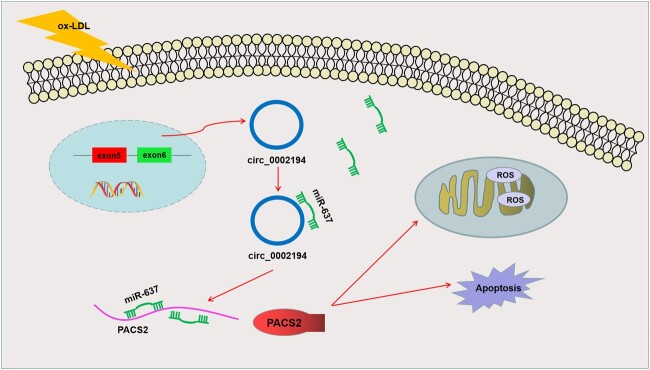
Schematic representation of circ_0002194 regulates cell apoptosis and oxidative stress by the microRNA-637/phosphofurin acidic cluster sorting protein 2 axis in oxidized low-density lipoprotein-treated endothelial cells. Circ: circular; miRNA-637: microRNA-637; OX-LDL: oxidized low-density lipoprotein; PACS2: phosphofurin acidic cluster sorting protein 2; ROS: reactive oxygen species.

Circ_0002194 acted as an miR-637 sponge to induce upregulation of PACS2, thus accelerating cell apoptosis and oxidative stress in ox-LDL-treated endothelial cells (Fig. 7).

## DISCUSSION

In the current study, circ_0002194 was shown to aggravate ox-LDL-induced endothelial cell apoptosis and oxidative injury by regulating the miR-637/PACS2 axis.

The dysfunction of endothelial cells is a pivotal event in the pathogenic progression of AS [3]. Dysregulated circRNAs have participated in the pathobiology to act as crucial regulators in AS. For instance, circ_0001445 was downregulated, and the upregulation of its expression suppressed cell proliferation in ox-LDL-treated HUVECs [16]. The silence of circ_0003204 inhibited the ox-LDL-mediated oxidative lesions and inflammatory response in HUVECs [17]. Circ_0002194 was more stable than linear transcripts in HUVECs, and ox-LDL upregulated the level of circ_0002194. Circ_0002194 has been reported to promote endothelial cell inflammation in an ox-LDL-induced AS model [10]. However, other biological functions of circ_0002194 in endothelial cells remain to be explored. Our data suggested that treatment of ox-LDL reduced cell viability and angiopoiesis but enhanced cell apoptosis and oxidative injury, whereas this damage was reversed with the downregulation of circ_0002194 in HUVECs. All in all, circ_0002194 promoted endothelial cell dysfunction of AS in vitro.

Then, we found that miR-637 levels were reduced in ox-LDL-treated HUVECs and that circ_0002194 interacted directly with miR-637. CircRNAs are known as natural sponges of miRNAs to inhibit the miRNA function in cardiovascular diseases [18]. Circ-0044073 was involved in the development of AS via the sponge effect on miR-107 [19]. Circ_0093887 prevented the dysfunction of endothelial cells by binding to miR-876-3p [20]. Also, our data showed that circ_0002194 regulated ox-LDL-induced cell injury by serving as the miR-637 sponge.

Noncoding miRNAs can regulate the biological processes of endothelial cells involved in the pathogenesis of AS [21]. Qun *et al.* discovered that miR-27b inhibited angiogenesis in endothelial cells and plaque stability in AS through downregulating Naa15 [22]. Huang *et al.* stated that miR-652-3p promoted the progression of AS and repressed endothelial repair by targeting CCND2 [23]. In this study, PACS2 was found to act as a target for miR-637, and miR-637 inhibition exacerbated the endothelial injury evoked by miR-637 via increasing the expression of PACS2.

PACS2 is associated with Ca^2+^ transfer in mitochondria, and it has been involved in the regulation of cardiovascular diseases. For example, PACS2 contributed to cardiomyocyte apoptosis in myocardial infarction and heart failure [24, 25]. The reversal of PACS2 knockdown for the regulatory function of the miR-637 inhibitor showed that PACS2 was partly responsible for ox-LDL-triggered endothelial injury, which was inconsistent with the results of the previous study [13]. More importantly, circ_0002194 was found to regulate the PACS2 level by targeting miR-637. The pathogenic function of circ_0002194 has been shown to be associated with the miR-6873-3p/MyD88 axis [10]. In contrast. we showed that circ_0002194 acted in endothelial cells by miR-637-mediated PACS2 upregulation.

In humans, circ_0002194 upregulation might be used for early screening and clinical diagnosis of patients with AS. In addition, circ_0002194 accelerated endothelial injury in an AS cell model by the miR-637/PACS2 axis. Nanotechnology can be used to knock down the expression of circ_0002194, which may target the miR-637/PACS2 axis to inhibit the progression of AS in patients. These detailed clinical manifestations require more exploration.

## CONCLUSION

Circ_0002194 interacted with miR-637 to induce the elevated expression of PACS2, which resulted in oxidative and apoptotic damage to endothelial cells treated with ox-LDL. Circ_0002194/miR-637/PACS2 was identified as a novel molecular network in the endothelial injury of AS.

## Data Availability

All relevant data are within the manuscript and its supporting information files.
